# Combined Use of Ionic Liquid-Based Aqueous Biphasic Systems and Microfluidic Devices for the Detection of Prostate-Specific Antigen

**DOI:** 10.3390/bios13030334

**Published:** 2023-03-02

**Authors:** Filipa C. Flora, Sofia B. Relvas, Francisca A. e Silva, Mara G. Freire, Virginia Chu, João Pedro Conde

**Affiliations:** 1 Instituto de Engenharia de Sistemas e Computadores-Microsistemas e Nanotecnologias (INESC MN), 1000-029 Lisbon, Portugal; 2 CICECO—Instituto de Materiais de Aveiro, Departamento de Química, Universidade de Aveiro, 3810-193 Aveiro, Portugal; 3 Department of Bioengineering, Instituto Superior Técnico, Universidade de Lisboa, 1049-001 Lisbon, Portugal

**Keywords:** microfluidics, prostate-specific antigen, human serum, aqueous biphasic systems, ionic liquids, extraction

## Abstract

Prostate cancer (PCa) is one of the cancer types that most affects males worldwide and is among the highest contributors to cancer mortality rates. Therefore, there is an urgent need to find strategies to improve the diagnosis of PCa. Microtechnologies have been gaining ground in biomedical devices, with microfluidics and lab-on-chip systems potentially revolutionizing medical diagnostics. In this paper, it is shown that prostate-specific antigen (PSA) can be detected through an immunoassay performed in a microbead-based microfluidic device after being extracted and purified from a serum sample through an aqueous biphasic system (ABS). Given their well-established status as ABS components for successful bioseparations, ionic liquids (ILs) and polymers were used in combination with buffered salts. Using both IL-based and polymer-based ABS, it was demonstrated that it is possible to detect PSA in non-physiological environments. It was concluded that the ABS that performed better in extracting the PSA from serum were those composed of tetrabutylammonium chloride ([N_4444_]Cl) and tetrabutylphosphonium bromide ([P_4444_]Br), both combined with phosphate buffer, and constituted by polyethylene glycol with a molecular weight of 1000 g/mol (PEG1000) with citrate buffer. In comparison with the assay with PSA prepared in phosphate-buffered saline (PBS) or human serum in which no ABS-mediated extraction was applied, assays attained lower limits of detection after IL-based ABS-mediated extraction. These results reinforce the potential of this method in future point-of-care (PoC) measurements.

## 1. Introduction

Cancer is a leading cause of mortality and it is one of the major public health problems worldwide [1]. Prostate cancer (PCa) is the most common non-cutaneous cancer in the west and the second most common cause of cancer-related mortality in males globally [2]. Therefore, independently of its indolent or aggressive nature, it is extremely important that reliable, accurate and validated cancer biomarker tests are developed to facilitate early diagnosis and treatment [1,3].

Prostate-specific antigen (PSA) is an androgen-regulated serine protease that belongs to the kallikrein family (kallikrein-related peptidase 3) [4,5]. PSA is produced in the prostate ductal and acinar epithelium, and it is secreted into the lumen [5]. Its function is to cleave semenogelins present in the seminal coagulum [5]. PSA is also important in oncology, serving as a cancer biomarker [5]. In fact, PSA was approved by the United States Food and Drug Administration (FDA) to monitor this disease, having been accepted for cancer detection [6]. Accordingly, the measurement of blood PSA levels can be a useful procedure to support PCa diagnosis [5]. Generally, concentrations in serum above the cut-off value of 4 ng/mL suggest that the patient has PCa, indicating the need to undergo biopsy procedures [5]. However, PSA blood levels are age dependent and also related to other factors, such as benign prostatic hyperplasia (BPH) and prostatitis, causing the occurrence of false positives when diagnosing PCa [7]. Moreover, it is not possible to distinguish between benign and malign tumors since both elevate serum PSA concentrations [8]. Yet, PSA is still the most common biomarker used for the detection of PCa. Indeed, over the decades, there was an improvement in PCa diagnosis rate specifically due to the implementation of the PSA test [2]. Nonetheless, there is still the need to improve the specificity of this test in order to accurately diagnose PCa and avoid over-diagnostics [7].

The PSA test is clinically performed using antibody-based techniques, namely microtiter plate enzyme-linked immunosorbent assay (ELISA) [9]. ELISA is a well-known method based on antigen–antibody recognition [9]. This test is highly sensitive and selective, easy to perform and has a high efficiency [10]. However, these assays have long incubation times, require the use of high-volume samples and reagents, bulky equipment, and skilled personnel [11]. Microfluidic systems, usually called lab-on-a-chip (LOC) devices, are an emerging technology that has great potential in miniaturizing and automating macroscale procedures [11]. Microtechnology brings several advantages in comparison with the usual large-scale protocols, such as a decrease in sample and reagent consumption, assay time, and costs, which allows faster analysis and detection [11]. Moreover, microfluidic devices are known to be portable and fit-for-purpose, can be embedded in other systems, and allow higher-throughput analysis [11]. Therefore, microfluidic-based biosensors are promising tools to achieve point-of-care (PoC) measurements that can, particularly, contribute to diagnose PCa.

Biomarkers are usually collected from biological fluids, serum, urine, saliva, etc. In particular, serum is a complex sample composed of a variety of proteins and other components. For instance, albumin is the most abundant serum protein, being present at a concentration varying from 35 to 50 mg/mL [12], followed by immunoglobulin G that can be found in concentrations ranging from 5.6 to 18 mg/mL [13]. These proteins can impact biomarker detection by leading to non-specific interactions, which may generate an increase in the background signal. Furthermore, biomarkers present in biological fluids usually appear in lower concentrations than the other components, and do not have the capability to replicate themselves. Thus, it is critical to extract and purify target biomarkers from serum and other biological fluids for an accurate detection.

Aqueous biphasic systems (ABS), primarily described in Albertsson et al. [14], allow promising results regarding the extraction and purification of molecules from biological samples. ABS are most commonly based on two polymers, two salts, or a polymer and a salt, which, if correctly selected, originate a biocompatible extraction method [15]. Above a certain concentration of each component, the solutions separate into two aqueous, yet immiscible phases, enabling the partition of target analytes [15]. This partition is dependent on the polarity, hydrophobicity, affinity to the phase and electrical charge of the components [15]. Even though polymer-based ABS are suitable for separation purposes, they present a limited polarity range, restricting their applicability in the complete extraction and purification of target analytes, especially from complex samples [16]. Ionic liquid-based (IL-based) ABS, firstly introduced by Gutowsky et al. [17], are among the best alternatives to replace polymer-based systems due to their enhanced structural tunability. Given the numerous anion and cation combinations accessible for the preparation of ILs, their properties can be modulated to provide a wider polarity window [18] and, in particular, milder conditions to intactly and completely extract proteins (such as PSA) [19]. IL-based ABS combine the benefits of ILs and ABS, possibly presenting advantages that traditional ABS cannot match. Depending on a correct formulation of the IL-based ABS, such advantages may include shorter separation time, lower viscosity, and higher extraction performance and/or selectivity [16,18]. Therefore, IL-based ABS have been widely used in biotechnological extraction and purification processes of proteins [19,20], including enzymes [21] and antibodies [22,23], as well as nucleic acids [24]. Besides these applications, Pereira et al. [25] demonstrated the ability of IL-based ABS as an effective method for the pre-treatment and concentration of cancer biomarkers from human urine to improve cancer diagnosis.

Reports on the use of microfluidic immunoassays to detect PSA are currently available. For instance, in 2015, Madaboosi et al. [9] presented an antibody-based sandwich immunoassay in a bare microfluidic channel using an amplification strategy based on biotin–streptavidin chemistry to reach clinically relevant limits of detection for PSA samples spiked in phosphate-buffered saline (PBS). Moreover, in the same year, Jolly et al. [7] demonstrated the use of a sandwich immunoassay for the detection of PSA in PBS, in which the capture antibody is replaced by a DNA aptamer. Nevertheless, these studies do not present a solution for the possible interference of biological matrices in the detection of PSA. A promising strategy to deal with some of the challenges of analyzing PSA in human fluids was proposed by Pereira et al. [26], reporting an IL-based ABS technique with the goal to extract and concentrate PSA from urine prior quantification by chromatography. Despite the advantages of microfluidic devices to expedite detection and of IL-based ABS to improve target analyte extraction, their combined use for PSA detection in human serum has lagged behind.

The aim of this paper is to demonstrate that it is possible to perform a microfluidic biomarker detection assay after carrying out an IL-based ABS-mediated extraction from a biological sample, thus opening the possibility of synergistically combining the advantages of the two approaches in the quantification of medical biomarkers. To fulfil this goal, both polymer-based and IL-based ABS are used to perform the extraction and purification of PSA from serum, which is then detected in a microfluidic device through an immunofluorescent assay. This work presents the initial results of the impact of ABS on biomarker extraction and the subsequent detection in exotic systems, such as ILs and polymers, showing new possibilities at the micro-scale and paving the way for future research of cancer diagnosis in non-physiological environments.

## 2. Materials and Methods

### 2.1. Fabrication of PDMS Microchannel Structures

The microfluidic structures were fabricated using soft lithography techniques. The hard mask, master mold and PDMS device fabrication were adapted from the literature as described in Pinto et al. [27]. A hard mask of patterned aluminum on glass is fabricated with standard microfabrication techniques. Since the microfluidic structure has two different heights, it was necessary to fabricate two different hard masks each containing the features of the required heights. The master mold was fabricated in two steps. After the cleaning of silicon substrates (University Wafer, South Boston, MA, USA), a SU-8 2015 negative photoresist (Microchem Corp., Newton, MA, USA) was spin coated (Laurell Technologies Corp., North Wales, PS, USA) to achieve thickness of 20 μm. After the pre-exposure bake (Hotplate, Stuart, Staffordshire, UK) at 95 °C for 4 min, the hard mask containing the 20 μm features was placed on top of the silicon substrate and the SU-8 was exposed to UV light (UV Light Technology Limited, Birmingham, UK) resulting in the printing of the 20 μm features. Then, a post-exposure bake at 95 °C for 5 min was perform followed by the development of the photoresist using PGMEA (Propylene glycol methyl ether acetate, Sigma-Aldrich, St. Louis, MO, USA). The substrate with the 20 μm features is again submitted to a similar procedure, using SU-8 50 (Microchem Corp., Newton, MA, USA), to pattern the 100 μm layer. In that case, the pre-exposure bake was done for 10 min at 65 °C followed by 30 min at 95 °C. In the pos-exposure bake, the substrate was baked for 1 min at 65 °C, the temperature was then increased until 95 °C and the mold was left for further baking for 10 min. A final baking step at 150 °C for 15 min was performed after each SU-8 layer fabrication to harden the features. The structures were fabricated using a mixture of curing agent with polydimethylsiloxane (curing agent KIT and Sylgard 184 PDMS, Dow Corning, Midland, MI, USA) at a weight ratio of 1:10. The master mold is taped to a PMMA frame and PDMS is poured on top of it and cured in the oven (Memmert, Schwabach, DE, USA) for 90 min at 70 °C. After curing, the PDMS is separated from the mold and the inlets and outlets were opened using 20 and 18 ga needles (Instech Laboratories, Inc. Plymouth Meeting, PA, USA), respectively. The sealing of the structure against a 500 μm PDMS membrane was by exposing both surfaces to an oxygen plasma in a plasma cleaner (Harrick Plasm, Ithaca, NY, USA) at high power for 60 s to oxidize both surfaces and, thus, form irreversible covalent bonds. Therefore, the fabrication of the PDMS structures take approximately 3 h 30 min as stated by Pinto et al. [27]; however, with more master molds, several devices can be fabricated simultaneously. Since each device consists of 30 single-use microchannels, it can be used 30 times. Regarding the chip’s lifetime, this is not restricted as long as the new structure is stored at room temperature in a clean environment.

**Figure 1:**
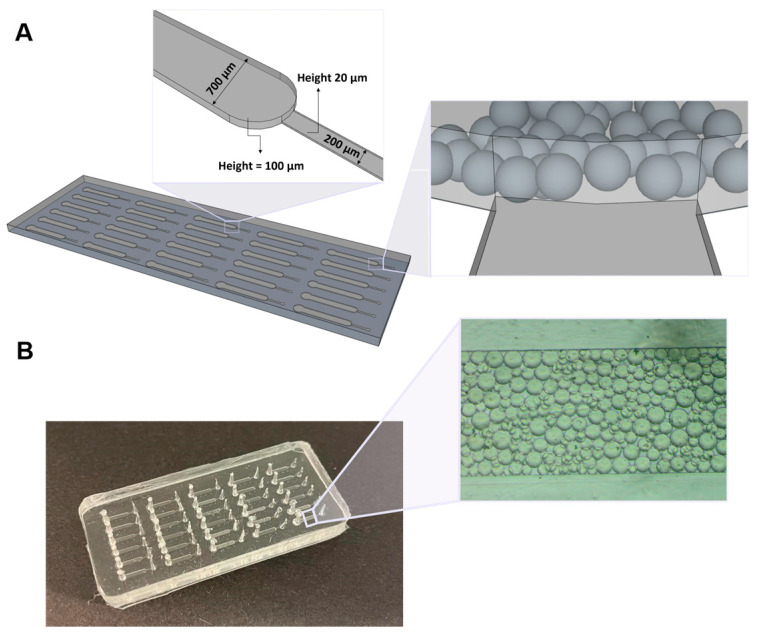
Microfluidic device used for the detection of PSA. (**A**) Schematics of the microfluidic structure comprising 30 microchannels with two sections, in which the first consists of a column with 700 μm width and 100 μm height, and the second has 200 μm width and 20 μm height. Representation of the microbeads trapped inside the main channel of the microfluidic chip, due to the different height of the two channels constituting the chip. (**B**) PDMS structure showing Protein G microbeads packed inside one of the microfluidic columns.

As shown in Figure 1, the final PDMS device consisted of 30 microchannels with two sections. The first section is a main channel with 700 μm of width and 100 μm of height, whereas the smaller section presents 200 µm width and 20 µm height, allowing the flow of the solutions towards the outlet. Since the Protein G microbeads used in this work have an average size diameter of 90 µm and due to the different heights of the two channels constituting the chip, the beads get trapped in the highest microfluidic column, because the 20 μm heigh of the smaller channel prevents them from reaching this area.

### 2.2. Reagents

PSA, human serum from male AB plasma and bovine serum albumin (BSA) were acquired from Sigma Aldrich. Phosphate-buffered saline (PBS) was prepared through the dilution of a PBS 10× stock solution (ThermoFisher Scientific, Waltham, MA, USA) in Milli-Q water to a final solution of 1× (pH 7.4, 25 °C). The salts, polymers, and ILs used to prepare the aqueous solutions and ABS are described in Table 1. Protein G Sepharose^®^ 4 fast flow beads were purchased from Cytiva, Marlborough, MA, USA. The anti-PSA mouse monoclonal capture (MABX5532) and detection (MABX5523) antibodies were acquired from Sigma Aldrich, St. Louis, MO, USA. The detector anti-PSA antibody was conjugated to the amine reactive dye Alexa Fluor^®^ (A430) NHS ester from ThermoFisher Scientific, Waltham, MA, USA to enable fluorescent detection. All aqueous solutions were prepared using PBS unless otherwise stated.

### 2.3. Microfluidic Handling

The microfluidic assays were performed using a NE-1002X syringe pump (New Era Pump Systems, Inc., Nassau, NY, USA). The 1 mL syringes (U-100 CODAN, Lensahn, Germany) were placed in the syringe pump and connected to a 20 ga luer stub adapter and a polyethylene BTPE-60 tube (Instech Laboratories, Inc., PA, USA). An open metallic adapter (Instech Laboratories, Inc., Montgomery, PA, USA) allowed the connection between the end of the BTPE-60 tube and the outlet of the microchannel. The method used to manipulate the fluids inside the channel consisted of pulling the solutions from the inlet towards the outlet by applying a negative pressure using the syringe pump. However, due to the inherent viscosity of some solutions, namely PEG1000 and PEG2000, to facilitate the correct flow of these components inside the microcolumn and prevent clogging of the chip, the pumping system was switched to pushing mode.

### 2.4. Microfluidic Immunoassay for the Detection of Spiked Solutions of PSA in PBS, Human Serum and Aqueous Solutions of ABS Components

[C_4_C_1_im]Cl, [C_4_C_1_pyrr]Cl, [N_4444_]Cl, [P_4444_]Cl, [P_4444_]Br, [Ch]Cl, PEG1000 and PEG2000 were prepared in PBS at 5 wt%. BSA was prepared from a stock solution at a concentration of 1 mg/mL and diluted in PBS to a 4% (*w*/*v*) solution. The capture and detector anti-PSA antibodies were used at a concentration of 100 µg/mL and PSA was spiked in PBS, human serum and several ionic liquids and polymers present in Table 1 at a final concentration of 25 ng/mL. In order to detect PSA spiked in PBS, human serum and different aqueous solutions of ABS components, a bead-based sandwich immunoassay was performed inside a microfluidic channel. The assay started with Protein G microbeads being pulled inside the microchannel at 5 µL/min, followed by a washing step with PBS for 2 min at 5 µL/min. Subsequently, the capture anti-PSA antibody was immobilized on the beads, followed by the PSA spiked solutions, then flowing BSA 4% (*w*/*v*) as blocking agent and finally the detector anti-PSA antibody. The capture and detector anti-PSA antibodies bind specifically to different epitopes of PSA, avoiding competition between them. In between every individual assay step, PBS was flowed through the microchannel to remove any unbound molecules, in a process known as intermittent washing. Unless stated differently, the reagents used were followed at a rate of 0.5 µL/min for 10 min and the PBS for rinsing at 5 µL/min for 1 min, as shown in Table 2.

### 2.5. Aqueous Biphasic System Preparation

To prepare the ABS, two buffers were used: phosphate and citrate buffer. ABS was obtained by adding the appropriate amounts of ILs or PEG, phosphate buffer solution (at 40 wt% of di-potassium hydrogen phosphate + potassium dihydrogen orthophosphate, pH 7) or citrate buffer solution (at 50 wt% of potassium citrate tribasic + citric acid, pH 7) (according to the buffer used in each system), human serum spiked with PSA at a concentration of 25 ng/mL and water to make the total weight of the system 100 wt%. The ILs used for the ABS-mediated extraction of PSA were [N_4444_]Cl and [P_4444_]Br and the PEGs were PEG1000 and PEG2000. When phosphate buffer was used as the salt component, the system was composed of 30 wt% IL or PEG + 12 wt% phosphate buffer + 10 wt% human serum + 48 wt% water, while when citrate buffer was used as the salt component, the system was composed of 25 wt% IL or PEG + 20 wt% citrate buffer + 10 wt% human serum + 45 wt% water. All systems were mixed using a vortex and centrifuged (mySPIN 12 Mini Centrifuge, ThermoFisher Scientific, Lisbon, Portugal) for 15 min at 2000 rpm. The mixture was left to settle for 10 min to allow the complete separation of the top and the bottom phases, with the appearance of a solid interphase being noticed. To minimize any contamination from the interphase, the bottom phase was first carefully collected using a 1 mL syringe touching the Eppendorf^®^ walls. The top phase was then collected following the same procedure. In these systems, the top phase is IL or PEG rich, while the bottom phase is salt rich. Control samples were also prepared following the same ABS preparation protocol, replacing the human serum spiked with PSA by non-spiked human serum solution.

### 2.6. On-Chip Microfluidic Immunoassays Following ABS-Mediated Extraction

After the ABS-mediated extraction of PSA, the analyte detection was accomplished through a bead-based sandwich immunoassay similar to that described in Section 2.3 performed for the top and bottom phases of each system. Briefly, the assay started with Protein G microbeads being packed inside the microfluidic column at 5 μL/min, followed by a washing step with PBS at 5 μL/min for 2 min. Next, the capture anti-PSA antibody was immobilized on the beads at 0.5 μL/min for 10 min. Subsequently, the top phase (IL- or PEG-rich phase) or the bottom phase (salt-rich phase) was passed through the microchannel at 0.5 μL/min for 10 min, followed by BSA 4% (*w*/*v*) as blocking agent and finally the detector anti-PSA antibody. In between every individual assay step, PBS was flowed through the microchannel at 5 μL/min for 1 min to remove any unbound molecules, in a process known as intermittent washing. The same protocol was followed for both phases of the control systems, where no PSA is present.

### 2.7. Image Acquisition and Analysis

The fluorescent images were acquired using an Olympus Microscope coupled with a XC30 digital camera. The fluorescence signal from the microchannels were obtained with an exposure time of 2 s and no optical gain. All the images were analyzed using the ImageJ software (National Institutes of Health, Bethesda, MD, USA). In these measurements, only the green channel from each RGB image was considered. The values correspond to the average of two independent experiments, always considering the same region of interest in the interior of the microchannels, after subtracting the background signal.

## 3. Results and Discussion

### 3.1. PSA Detection in Microfluidics

**Figure 2:**
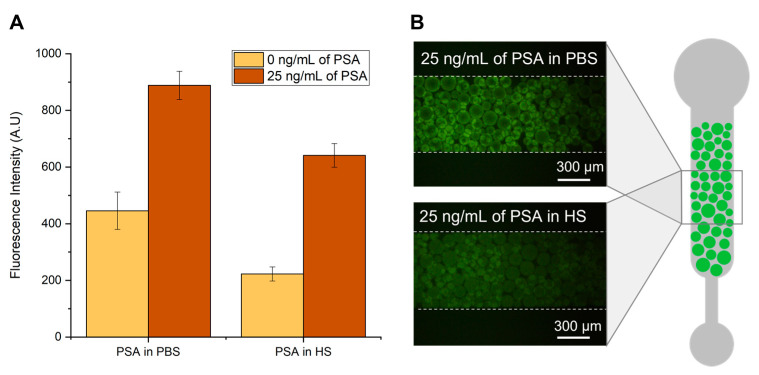
Detection of PSA in phosphate-buffered saline (PBS) and human serum (HS). (**A**) Fluorescence intensity from bead-based sandwich immunoassay performed in the presence (25 ng/mL) or absence (0 ng/mL) of PSA spiked in PBS or undiluted HS. (**B**) Fluorescence microscopy of Protein G agarose beads packed on a PDMS microchannel after flowing the detector labelled antibody in the presence of PSA spiked in PBS (top image) or in HS (bottom image). All images were contrast enhanced for visualization purposes. The error bars represent the standard deviation between two independent experiments.

Considering that most biomarkers are present in biological fluids, the use of a microfluidic device for the diagnosis of PCa would require measurements of PSA in real human serum samples. Therefore, the influence of human serum in the detection of PSA was first assessed, and the results are depicted in Figure 2.

Figure 2 shows the influence of human serum on the fluorescence intensity derived from a bead-based PSA sandwich immunofluorescent assay. Capture anti-PSA antibodies are immobilized on Protein G agarose beads, and the captured PSA is detected with fluorescently labelled anti-PSA antibodies, as detailed in the Materials and Methods section. The experiments were performed with an undiluted concentration of human serum and compared with PBS spiked with the same concentration of PSA. It is possible to observe an overall decrease in the fluorescence signal when the detection of PSA is performed in serum compared to PBS. This signal reduction was similar to that reported earlier [28] and is attributed to the complex nature of biological matrices. Serum samples present high concentration of proteins, in which albumin accounts for almost 80% of its content. The highly abundant proteins in serum can bind non-specifically to the remaining assay molecules, creating a blocking effect and reducing the fluorescence signal. Another possible explanation is that the viscosity inherent to increased concentrations of serum proteins in solution may pose constraints to the diffusion of molecules inside the microfluidic column, consequently impairing the capture and detection of PSA molecules.

From Figure 2 it is also possible to observe the considerable amount of fluorescence signal in the absence of PSA for both experiments performed in PBS and human serum. This might be caused by non-specific molecular interactions resulting from non-specific affinity of the detector antibody to the Protein G beads or to the capture antibody. However, there is still a significant amount of specific signal obtained in the presence of 25 ng/mL of PSA for both PBS and human serum samples. Nevertheless, these results highlight the need to extract and purify PSA from the serum sample for a more efficient detection.

### 3.2. PSA Detection in Aqueous Solutions of ABS Components

Prior to the introduction of a PSA extraction and purification step mediated by ABS, the influence of aqueous solutions of commonly used ILs and polymers in the molecular recognition of PSA was assessed. IL-based ABS have been used as alternatives of polymer-based systems for the extraction of proteins and enzymes with several studies reporting higher extraction efficiencies in comparison with commonly used PEG-based ABS [29,30,31,32]. In addition to their wide usage incidence in ABS preparation, the ILs selected are representative of five distinct families and have been widely exploited regarding their ability to extract and purify a different range of biomolecules [19,20,26,33,34,35,36,37]. Moreover, the ILs selected allow investigating a broad range of polarities, while the chosen PEGs are the most used polymers for ABS components and differ on molecular weight. This assessment helps identifying ABS components compatible with the detection method, which can be further used in the combined off-chip extraction and detection studies.

The bead-based sandwich immunofluorescent assay was performed for the quantification of PSA spiked in six ILs and two different molecular weight PEGs aqueous solutions. The signals obtained were compared with the fluorescence intensity previously obtained with the spiked samples prepared in PBS. The results are shown in Figure 2. For each system, PSA samples with concentrations of 25 and 0 ng/mL were spiked in final concentrations of PBS 1×, 5 wt% IL solutions or 5 wt% PEG solutions.

**Figure 3:**
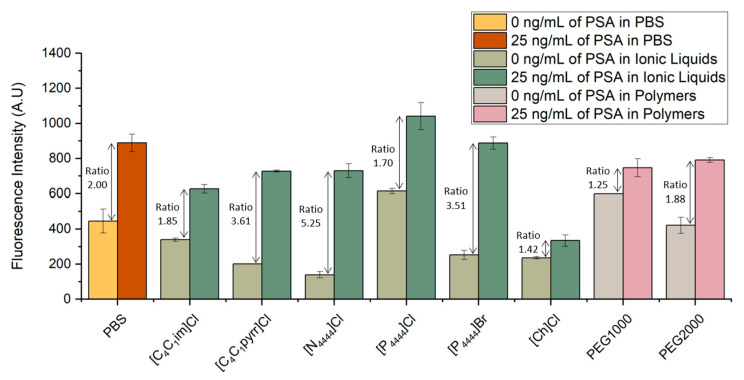
Influence of different ILs and polymer solutions in the detection of PSA. Fluorescence intensity from bead-based sandwich immunoassay performed in the presence (25 ng/mL) or absence (0 ng/mL) of PSA spiked in PBS, six IL solutions ([C_1_C_4_im]Cl, [C_1_C_4_pyrr]Cl, [N_4444_]Cl, [P_4444_]Cl, [P_4444_]Br, and [Ch]Cl) and two PEG solutions with different molecular weight (1000 g/mol and 2000 g/mol). The error bars represent the standard deviation between two independent experiments.

Figure 3 validates the possibility of detecting PSA in several ILs and PEG aqueous solutions. Looking at the signal to noise ratio, defined as the ratio of the fluorescent signal from the samples spiked with 25 ng/mL of PSA to that of control samples with no spiked PSA concentration, one sees that all IL- and PEG-based systems tested were able to reach a ratio similar to or even higher than the ratio obtained in the spiked samples prepared in PBS. In particular, [C_1_C_4_pyrr]Cl, [N_4444_]Cl and [P_4444_]Br proved to be the ILs presenting lower levels of interference in the molecular recognition of PSA, as shown by the higher signal-to-noise ratio in comparison even with the samples spiked in PBS. These higher ratios are related with a decrease in the fluorescence signal obtained from the control samples, translating into a reduction in non-specific interactions. This observation can be explained by the fact that the ILs act as a blocking agent due to their constituents and chemical properties, minimizing the interference of the detector antibody with the remaining non-target molecules. Regarding the systems prepared with PEG1000 and PEG2000, the latter presents a higher signal-to-noise ratio, with the control sample reaching lower values of fluorescence in comparison to PEG2000. This ratio is also very similar to the one obtained for the systems prepared in PBS. These observations confirm that the detection of PSA presents a comparable performance in PBS and in different chemical solutions, such as ILs or polymers. To the best of our knowledge, there have been no previous reports in the literature of immunoassay detection of PSA in IL or polymer solutions in microfluidics. These observations confirm the usability of ABS as biomarker extraction/purification options to overcome the effect of matrix interference.

### 3.3. Off-Chip ABS with Subsequent On-Chip Microscale PSA Detection

After the initial screening of different ILs and polymers to assess their interference in the detection of PSA, some of these components were selected for the formation of ABS to extract and purify PSA from spiked serum samples. The selection of the phase forming components was done according to the signal to noise ratios, achieved by the various ILs and polymers studied, as well as their behavior inside the microfluidic channel. As the salt component of ABS, the two widely employed phosphate (K_2_HPO_4_ + KH_2_PO_4_) and citrate (C_6_H_5_K_3_O_7_ + C_6_H_8_O_7_) buffers were applied to assure pH 7 and protein- friendly conditions [38,39].

**Figure 4:**
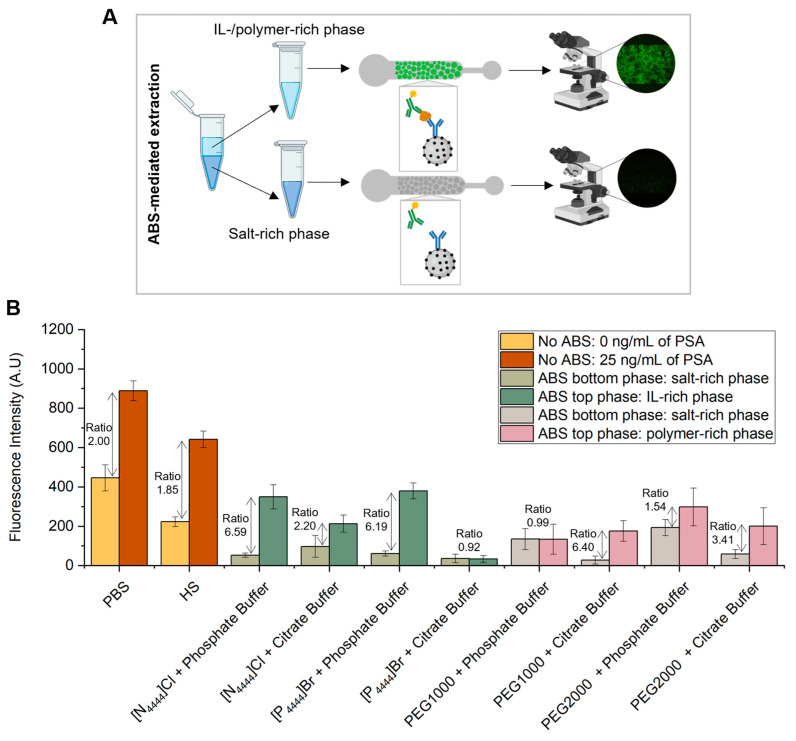
Detection of PSA with and without ABS-mediated extraction. (**A**) Phase separation during the off-chip ABS-mediated extraction, followed by on-chip detection of PSA. (**B**) Fluorescence intensity from bead-based sandwich immunoassay performed in the presence (25 ng/mL) and absence (0 ng/mL) of PSA spiked in PBS or HS without ABS-mediated extraction and with IL- and polymer-based ABS-mediated extraction with PSA spiked in HS. The measurements of fluorescence intensity obtained for the assays performed with ABS-mediated extraction were calculated by subtracting for each phase (top and bottom phases) the fluorescence intensity values from the control system with 0 ng/mL of PSA to the fluorescence intensity values from the system with 25 ng/mL of PSA to eliminate the effect of non-specific interactions from the detection assay. The error bars represent the standard deviation between two independent experiments.

ABS were shown to be formed in the presence of human serum in the following mixture compositions, which were further used to carry out the extraction of PSA: 30 wt% IL or PEG + 12 wt% salt + 10 wt% human serum + 48 wt% water, for systems prepared with phosphate buffer as salt component and 25 wt% IL or PEG + 20 wt% citrate buffer + 10 wt% human serum + 45 wt% water for systems prepared with citrate buffer as salt component. These two mixture compositions were chosen based on the phase diagrams of the respective IL–salt–water or polymer–salt–water ternary systems [40,41,42,43], ensuring a common biphasic point for each set of systems. For each system composition, two ABS were prepared: one with human serum spiked with 25 ng/mL of PSA and another with human serum with 0 ng/mL of PSA, corresponding to the control sample. After a careful separation of phases, the amount of PSA present in the top and bottom phase was quantified using a microfluidic bead-based sandwich assay, as previously described (Figure 4A). The results obtained were compared with the fluorescence signals derived from the assays performed with PSA spiked in PBS, in which no ABS-mediated extraction was used (Figure 4B). For each phase, the fluorescence signal obtained for the control sample, i.e., human serum sample with 0 ng/mL of PSA, was subtracted from the fluorescence signal obtained for the human serum spiked with 25 ng/mL of PSA. This calculation was done to eliminate the effect of non-specific interactions inherent to the sandwich immunoassay herein developed.

From Figure 4B it is demonstrated the partition of PSA towards the IL-rich phase, mainly due to a salting-out effect and in line with previous results [26]. Exceptions to this behavior are represented by the systems composed of [P_4444_]Br + citrate buffer and PEG1000 + phosphate buffer, in which the quantification of PSA is equal for both phases. This might be justified by potential PSA losses due to precipitation at the systems’ interphase. Among the two types of ABS under appraisal, the IL–salt and polymer–salt combination determines the extent of PSA partition to the top phase. While ILs achieve higher ratios in the presence of phosphate buffer, polymers perform better in the presence of citrate buffer. In addition to the salting-out effect, it seems that the PSA partitioning is controlled by a complex set of multiple and specific interactions between PSA and the ABS constituents. According to the ratios between top and bottom phases, the systems with higher levels of PSA present in the IL-rich phase are the ones composed of [P_4444_]Br + phosphate buffer, [N_4444_]Cl + phosphate buffer and PEG1000 + citrate buffer, which were selected for further immunoassay sensitivity studies.

The proposed method demonstrates the preferential partition of PSA to the IL- or PEG-rich phase, validating the usability of ILs and polymers as phase-forming components of ABS for the extraction and purification of target analytes in a complex sample matrix, such as human serum. In fact, immunoassays are often affected by matrix inference effects, due to the lower abundance of protein biomarkers in comparison with other non-target proteins present in these biofluidic matrices, which can impair the sensitivity of the detection assay. In the present work, the results presented show a successful PSA extraction using ABS without compromising the detection immunoassay itself. Moreover, these systems, in particular IL-based ABS, are advantageous due to their biocompatible nature, i.e., they ensure protein stability and biological activity, which might be compromised with traditional organic solvents.

### 3.4. Sensitivity of Sandwich Immunoassay for Detection of PSA Following ABS-Mediated Extraction

After selecting the most promising ABS to use during the PSA extraction step, a study was carried out to characterize the sensitivity of the detection method developed. Five sensitivity curves were obtained, as shown in Figure 4: two for systems without ABS-mediated extraction, with PSA spiked in PBS or human serum; and three with IL-based and PEG-based ABS-mediated extraction with PSA spiked in human serum.

**Figure 5:**
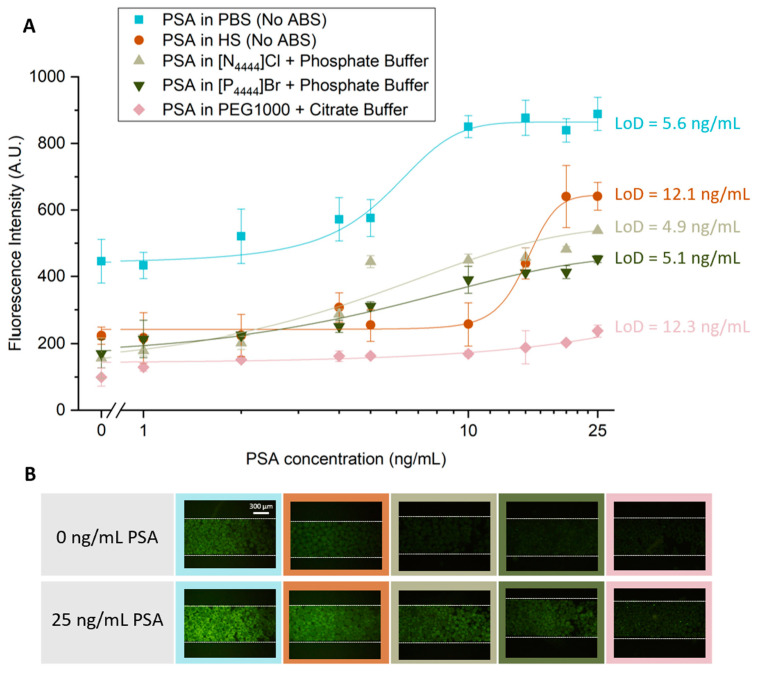
Sensitivity curves for increasing PSA concentration without ABS-mediated extraction and with IL- and PEG-based ABS-mediated extraction. (**A**) For the systems with no ABS-mediated extraction, the fluorescence intensity is derived from the bead-based sandwich immunoassay performed with solutions ranging from 0 to 25 ng/mL of PSA spiked in PBS or human serum. For the systems which had a IL- or PEG-based ABS-mediated extraction, the fluorescence intensity is derived from the bead-based sandwich immunoassay performed with solutions ranging from 0 to 25 ng/mL of PSA spiked in 10 wt% human serum which were added to three ABS systems composed of 30 wt% IL and 12 wt% phosphate buffer, in which the ILs used were [N_4444_]Cl and [P_4444_]Br and 25 wt% PEG1000 and 20 wt% citrate buffer. (**B**) Fluorescence microscopy of Protein G beads in the presence (25 ng/mL) and absence (0 ng/mL) of PSA spiked in PBS and HS for the systems in which no ABS-mediated extraction was used and spiked in HS for the systems where different compositions of ABS were used. All images were contrast enhanced for visualization purposes. The error bars represent the standard deviation between two independent experiments.

The response obtained for this sensitivity study follows a dose–response fitting and the limits of detection (LoDs) were calculated as

(1) $$\mathrm{LoD}={\;\mathrm{mean}}_{\mathrm{blank}}+3\sigma ,$$

in which the ${\mathrm{mean}}_{\mathrm{blank}}$ corresponds to the mean value of the blank sample (control sample with 0 ng/mL of PSA), and σ corresponds to the standard deviation of the blank sample, being represented in Figure 5 and Table 3

For all calibration curves obtained, there is an increase in fluorescence intensity with increasing concentrations of PSA. Higher values of fluorescence are achieved when there is no ABS-mediated extraction, and the PSA is spiked in PBS. However, this system also presents higher levels of non-specific molecular interactions. The LoD for this approach was 5.6 ng/mL of PSA, slightly above the clinical cutoff value of 4 ng/mL of PSA. Our group previously reported a LoD of 5 ng/mL of PSA using a similar sandwich immunoassay system performed in a bare microfluidic channel with target PSA spiked in PBS, in which it was used an amplification strategy to lower an initially obtained LoD of 21.4 ng/mL [9]. Therefore, the current results are comparable to those reported in the previous study. For the system where PSA was spiked in human serum but where no ABS-mediated extraction was performed, there is an overall reduction in fluorescence intensity, throughout all concentrations tested in comparison with the system with PSA spiked in PBS. However, it is possible to observe a reduction in the influence of non-specific interactions, due to the complexity of this biological fluid. Nonetheless, there is an increase in the LoD achieved with this approach to a value of 12.1 ng/mL. This value is comparable to a LoD of 10 ng/mL of PSA in human serum samples previously reported by Pinto and co-workers [27], in which a sandwich immunoassay performed with Protein A beads was used to study the detection of PSA within the clinically relevant window. Figure 5 also shows similar levels of fluorescence intensity for the two systems where IL-based ABS were used as extraction strategy, with a reduction in LoDs to approximately 5 ng/mL, slightly above the clinical threshold of 4 ng/mL, showing the efficacy of these systems in cleaning and extracting PSA. Finally, the LoD was evaluated using a PEG-based ABS to perform the PSA extraction. In this case, it is possible to observe a decrease in the overall fluorescence intensity presented by all samples in comparison with the remaining systems and an increase in the LoD to 12.3 ng/mL. These observations are explained by the increased viscosity associated to PEG solutions, hindering the diffusion of molecules inside the microfluidic column, which affects the molecular recognition of PSA.

As previously mentioned, Pereira et al. [26] reported the use of IL-based ABS as an effective extraction and concentration technique of PSA from human urine. Despite the claimed advantages of using urine to detect PSA to assist a differential diagnosis of prostate diseases [44], its clinical utility remains under development. The efficiency of IL-based ABS in the extraction of PSA from a more complex and clinically used matrix is here attested using human serum. Therefore, the possibility to reduce/eliminate matrix interference using IL-based ABS for the extraction of PSA in human serum is promising for the use of PoC devices.

## 4. Conclusions

In this paper, IL- and PEG-based ABS were successfully used to extract and purify PSA prior to detection using a bead-based sandwich immunoassay in a microfluidic device. The interference of a biological matrix in the detection of PSA was first assessed through an on-chip immunoassay, by comparing the fluorescence intensity of PSA spiked in human serum samples with PSA prepared in PBS. A clear matrix interference from human serum was observed and a target analyte extraction technique was deemed necessary to achieve a more accurate and reliable POC analysis of PSA. To achieve this, several ILs and PEGs, commonly used in ABS for extraction and purification purposes, were studied, appraising their interference in the detection of PSA. Furthermore, IL- and PEG-based ABS were tested and proved to be efficient in managing the effects of the complex biological matrix human serum. The platform developed for the detection of PSA using the most promising IL-based ABS as an extraction technique was able to reach LoDs of approximately 5 ng/mL. Considering the PSA clinical range of 4–10 ng/mL, this PoC system proved to be effective in achieving reliable results within the relevant range for PCa diagnosis.

Despite the demonstrated applicability of IL-based ABS-mediated extraction in supporting the accurate detection of PSA by a microfluidic device, there are some limitations that still need to be addressed to reach a full sample-to-answer approach. The strategy herein proposed required the use of a microscope for signal quantification. Instead, integrated optical detection could be achieved through the incorporation of photodetectors fabricated in house in a similar strategy followed by Novo et al. [45], comprising the addition of hydrogenated amorphous silicon (a-Si:H) photodiodes for data acquisition. In addition to sensors integration, on-chip control of fluidic handling can also be included in the microfluidic device through the integration of microvalves and micropumps, as previously performed by Pinto et al. [46].This approach would allow the system to perform more complex assays without increased user input. Finally, regeneration of the microfluidic chip can be considered, ensuring the reusability of each microcolumn with the same packing bed. This would considerably reduce the variability associated with the bead packing in each assay. A similar strategy was implemented by Caneira et al. [47] for microchips intended to perform DNA detection and Pinto et al. [46] for microchips developed for the optimization of chromatography operating conditions. It is therefore necessary to automate this detection platform to fully exploit its potential as a PoC device, which is still being researched.

## Figures and Tables

**Table 1:** Summary of all the salts, polymers and ILs tested.

Type of ABS Component	Abbreviation	Name	Supplier	Purity (%)
Salts	Citrate Buffer	Potassium citrate tribasic monohydrate Citric acid 1-hydrate for analysis, ACS, ISO	Acros Organics, Geel, Belgium and Panreac, Chicago, IL, USA	99 99.5–102.0
	Phosphate Buffer	Di-potassium hydrogen phosphate trihydrate Potassium dihydrogen orthophosphate	Scharlau, Barcelona, Spain and Fisher Chemical, Waltham, MA, USA	98–102 99.95
Polymers	PEG1000	Polyethylene glycol (1000)	Alfa Aesar, Haverhill, MA, USA	-
	PEG2000	Polyethylene glycol (2000)	Alfa Aesar, Haverhill, MA, USA	-
Ionic Liquids	[C_4_C_1_im] Cl	1-butyl-3-methylimidazolium chloride	Iolitec, Heilbronn, Germany	99
	[C_4_C_1_pyrr] Cl	1-butyl-1-methylpyrrolidinium chloride	Iolitec, Heilbronn, Germany	99
	[N_4444_] Cl	Tetrabutylammonium chloride	Sigma, St. Louis, MO, USA	≥97
	[P_4444_] Cl	Tetrabutylphosphonium chloride	Iolitec, Heilbronn, Germany	>95
	[Ch]Cl	Cholinium chloride	Acros Organics, Geel, Belgium	99
	[P_4444_] Br	Tetrabutylphosphonium bromide	Iolitec, Heilbronn, Germany	>95

**Table 2:** Summary of the flow rates and flow times for each solution used in the immunoassay and total assay time.

Solution	Q (uL/min)	t (min)
Protein G microbeads	5	~2
PBS	5	2
Anti-PSA capture Ab	0.5	10
PSA	0.5	10
BSA 4% (*w*/*v*)	0.5	10
Anti-PSA detector Ab–A430	0.5	10
PBS (intermittent washing)	5	1
Total assay time	~48 min	

**Table 3:** Sensitivity comparison between systems in which no ABS-mediated extraction was performed (PBS and HS) and with IL- and PEG-based ABS-mediated extraction.

System	Minimum Concentration of PSA Tested (ng/mL)	Data Correlation (R^2^) w/ Dose Response Fitting	LoD
PBS	1	0.9855	5.6
HS		0.9699	12.1
[N_4444_]Cl + phosphate buffer		0.9568	4.9
[P_4444_]Br + phosphate buffer		0.9944	5.1
PEG1000 + citrate buffer		0.9457	12.3

## Data Availability

The data that support the findings of this study are available upon reasonable request.
